# Understanding nutritional outcomes through gendered analysis of time-use patterns in semi-arid India

**DOI:** 10.1016/j.gfs.2019.04.001

**Published:** 2019-12

**Authors:** Ravula Padmaja, Soumitra Pramanik, Prabhu Pingali, Cynthia Bantilan, Kasala Kavitha

**Affiliations:** a Senior Scientist–Gender Research, International Crops Research Institute for the Semi-Arid Tropics, Patancheru, India; b Senior Scientific Officer, International Crops Research Institute for the Semi-Arid Tropics, Patancheru, India; c Professor and Head, TCI, Cornell University, Ithaca, USA; d Honorary Fellow, International Crops Research Institute for the Semi-Arid Tropics, Patancheru, India

**Keywords:** Nutritional outcomes, Time use, Gender, Agriculture, Semi-arid tropics

## Abstract

The objective of this paper is to understand individual nutritional outcomes through an examination of gendered time use patterns. The analysis of the data from eight villages in the Semi-Arid Tropics (SAT), India confirm previous conclusions about the gendered influence of agricultural interventions, especially time demands on the rural poor. Agricultural interventions in the harsh, drought-prone environment of the SAT tend to increase the time burden on women. Sociological perspectives indicate that changes in time use patterns are also due to changing agricultural practices such as cropping patterns, type of productive work (farm to non-farm) among other factors, leading to variations in outcomes like nutrition for different members of the household. These changes demand innovative gender-responsive approaches and policies to leverage agriculture for nutrition and health. The paper concludes that empowering women through more information and control over income, assets and resources enhances their agency to make decisions for efficient time use, food consumption, sanitation and healthy practices. The authors opine that the context is important and policies must be based on sound data and rigorous analysis, including social and gender considerations.

## Highlights

• A gendered analysis of time-use patterns in agriculture suggests time use a transformative indicator of gender equality.
• Age, education and social groups have varied effects on time-use patterns of men and women in SAT households.
• Rural diets to become more diverse and healthy through enhanced quality and quantity of the food baskets along with better sanitation practices.

## 1 Introduction

Agriculture, an engine of growth for reducing poverty, continues to be a dominant occupation in rural Asia and sub-Saharan Africa. There is growing recognition that women are now playing greater roles and making essential contributions to agricultural and rural economies in developing countries. They are often managing complex households and multiple livelihood strategies (FAO et al., 2011) in bringing about rural transformation in general and agricultural transformation in particular. Building on the articulation presented in the above mentioned report that roles of men and women vary considerably between and within regions, the authors reiterate that changes in the roles and responsibilities of women are dependent on prevailing social and cultural norms, agricultural production systems and the transformation of the rural economies, including social transformations. The activities of rural women also span the continuum from producing agricultural crops, participating in the non-farm sector through wage employment and small entrepreneurial efforts, livestock and allied activities, postharvest processing, marketing and trade, to collecting fuel and water, domestic activities including cooking, participating in the care economy of the household and in community activities and group action. There is an observed progressive feminization of agriculture (Binswanger-Mkhize, 2012; FAO, 2017), which optimistically presents opportunities for women to improve their agency and the well-being of their families, households and communities at large.

Nutrition is an essential aspect of well-being where agriculture has a direct connect and influence. Adequate nutritional attainment is “equally” essential for women and men, and boys and girls. Given this argument, there is an increasing focus on studying and understanding with greater clarity the linkages between agriculture and human nutrition with a gender lens, in order to identify nutrition-sensitive agricultural interventions. Literature identifies several pathways linking agriculture and nutrition, some of which specifically address women's time use and nutrition in agriculture (Herforth and Harris, 2014). A number of studies propose that increasing women's engagement in agriculture and allied activities contribute to child undernutrition in a number of ways, such as there being less time to participate in the care economy of the household, improper infant and child feeding practices, and the provision of cooking, fetching water and fuel wood; and health services to the household (Kadiyala et al., 2014; Headey et al., 2011; Mondiale, 2008; Glick, 2002; Smith, 2003). On the other hand, a number of studies demonstrate the pathways of impact of women's increased engagement in agriculture on positive household nutrition and food security outcomes (Quisumbing and Maluccio, 2003; Duflo and Udry, 2004; Doss, 2006; Qian, 2008; Sraboni et al., 2014; Malapit and Quisumbing, 2015). A summary of evidence documented by Van den Bold et al. (2013) demonstrates the important linkages between dimensions of women's empowerment and nutritional outcomes. This paper appeals to the definition of empowerment proposed by Kabeer (1999), who describes it as a process and as expanding people's ability to make strategic life choices, particularly in contexts in which this ability had been denied to them.

This paper contributes to the debate on agriculture, time use, gender and nutritional outcomes through an empirical analysis of micro-level data in selected villages in SAT India. It poses two questions:

• What is the share of women and men in time spent in the farm and household economy in the fragile environments of the Semi-Arid Tropics (SAT)?
• How does this impact their own nutritional status?

### 1.1 Agriculture, nutrition and time use: a succinct review of literature

Research on gender, nutrition and health in developing countries including India is drawing a lot of interest and attention. India has one of the highest underweight burdens in the world (42%), and is exhibiting steadily rising obesity and overweight trends. This has seen the emergence of the phenomenon known as the ‘triple burden’ of malnutrition – undernourishment, micronutrient deficiencies and obesity (Meenakshi, 2016; Pinstrup-Andersen 2007), more so in the fragile environments of SAT India where the sustainability of livelihoods is a major concern. In India, for example, smallholder farmers (possessing less than or 1 ha of land) are most likely to be malnourished, and among them, women, who in many cases are engaged in the majority of agricultural labor, are disproportionately likely to be malnourished (Herforth et al., 2012). There is abundant evidence of farmers being less productive when they are malnourished (Sahn, 2010; Haas et al., 1995); therefore, improving the nutritional status of rural populations will improve agricultural productivity in the first place and also child nutrition via the mother.

There is enough literature on the multifaceted and dynamic linkages between agriculture, health and nutrition (Headey, 2012; Kadiyala et al., 2014; Ruel and Alderman, 2013; Von Braun and Kennedy, 1994). Kadiyala et al. (2014) emphasize the role of gender division of labor in agriculture, which influences the time women take to care for themselves and young children, the intra-household allocation of food which affects women's nutritional status and its intergenerational effects on nutrition outcomes, and women's power in decision making which influences whether gains in income translate into nutritional improvement. Women's time constraints in a household impact food consumption patterns, thereby affecting the overall health and nutritional status of the household (Jabs and Devine, 2006).

The most recent systematic review of evidence on agriculture, time use and nutrition in rural areas in many low and middle income countries was conducted by Johnston et al. (2015, 2018). They show evidence that women play a key role in agriculture, and this is reflected in their time commitments to these activities, either as farm workers or farm managers. Their analysis also reveals that women are important actors in the uptake of and response to agricultural interventions which tend to increase time burdens on women, men and children. The authors infer that nutritional impacts vary with household responses to increased time burden and workload and suggest that the link between time burden and nutrition is complex. Johnston et al. (2018) provide new evidence that agricultural interventions may tend to increase time burdens of beneficiaries. Beatty et al. (2013), in the context of a Supplemental Nutrition Assistance Program (SNAP), explored the relationship between food security and time spent by women in food preparation and related activities, and found a significant relationship between these two. Although a causal relationship was not established, the results highlighted the relationship between time spent on food-related activities and program participation and call for policies that address households that face time and budget constraints. Johnston et al. (2018) in their review paper also conclude that while changing time use tends to change nutritional outcomes, the pathways are complex and there is no agreement on the impact. These evidences clearly identify time use as an important data and suggest that understanding gendered time use patterns helps in appreciating the different socio-economic contexts that go into gender and nutrition policies, program perspectives and recommendations (Hirway, 2009; Antonopoulos and Hirway, 2010).

There is limited empirical evidence (Beatty et al., 2013; Chen et al., 2015; Eswaran et al., 2013; Mothersbaugh et al., 1993; Peterman et al., 2013) to test and corroborate the linkages between time use and nutritional outcomes of individuals. This could be due to lack of micro-level data on the nutritional status of women and children, and women's time use in agriculture and domestic work.

Such evidence assumes great significance in the harsh, vulnerable environments of the semi-arid tropics (SAT) of India which are facing acute water shortage, continuous drought as well as a policy bias towards rainfed agriculture, in addition to the prevalence of rigid gender, social and cultural norms dictating women and male outmigration and progressive feminization of agriculture. Putting a nutrition lens on an agricultural investment/intervention can improve gender equity in that investment – an increasingly common goal of the agriculture sector – because it shifts the focus towards labor, income, asset control and time use of women. This paper attempts to understand the linkages between time use, agriculture and nutrition from a gender and diversity perspective, through a descriptive analysis, using data generated from qualitative and quantitative surveys in selected villages in the semi-arid tropics of India. The sociological descriptions are complemented with statistical analysis.

## 2 Methods

This paper uses data from two sources: Village Dynamics Studies in South Asia (VDSA)^1^ and a special purpose survey^2^ implemented in eight VDSA villages in 2013–14. The analysis presented is based on a sample of 462 households covering 1284 respondents in the 18–60 age group, of which 649 were male and 635 were female. The respondents belonged to eight locations in Telangana (then united Andhra Pradesh), Andhra Pradesh and Maharashtra states of India. The selection of sample villages, households and individuals for the VLS/VDSA are elucidated in Walker and Ryan (1990), Rao and Kumara Charyulu (2007) and Deb et al. (2014). To summarize, the six villages from Telangana and Maharashtra were selected based on the prevailing soil type, rainfall and cropping pattern in the region during the 1970s. The sample was selected based on the land holding, and an equal number of households (10) from large, medium and small land holdings were selected together with households with no land. Thus, initially 40 households were selected from each village in 1974–75. Over the years, the split-off and spin-off households that belong to the parent households were also part of the study; thus the sample size increased accordingly (see Box 1). While the head of the household was interviewed, it was ensured that the spouse was also present and her response was also sought. Data was collected by resident investigators every month using a standardized questionnaire and from the same households and individuals from 1975 to 84. Between 2001 and 2004, the frequency of data collection from the same households was either annual or bi-annual based on funding availability. Starting 2005–2014, monthly data collection was resumed, similar to 1975-84^3^.

Box 1 **Study locations, survey period and variables used for analysis.**

**Table:** 

State in India	Villages	VLS/VDSA surveyed year	Sample size (number of households) as of 2014	Variables from the VDSA	Variables from Special purpose survey conducted in 2013–2014
Telangana (Mahabubnagar district)	Aurepalle	1975–84, 2001–2014	68	1. Socio-economic variables such as age, gender, and education of household members. 2. Consumption expenditure pattern of the household. 3. Cultivation details such as input and output values 4. Cropping pattern	1. Time use (quarterly) 2. Dietary diversity (quarterly) 3. Anthropometry (height and weight of the individuals in the household, one time for adults; quarterly for children) 4. Dwelling characteristics (one time) 5. Sanitation facilities (half yearly) 6. Source of drinking water (half yearly)
	Dokur	1975–84, 2001–2014	46		
Maharashtra (Akola district)	Kanzara	1975–84, 2001–2014	64		
	Kinkhed	1975–84, 2001–2014	51		
Maharashtra (Solapur district)	Shirapur	1975–84, 2001–2014	89		
	Kalman	1975–84, 2001–2014	72		
Andhra Pradesh (Prakasam district)	Pamidipadu	2009–2014	42	1. Socio-economic variables such as age, gender, and education of household members. 2. Consumption expenditure pattern of the household. 3. Cultivation details such as input and output values	
	JC Agraharam	2009–2014	42

Alt-text: Box 1

The aim of the special purpose survey during 2013–14 was to understand the nutritional status of the men, women and children of these households as well as the empowerment of men and women in the harsh, fragile environments of the SAT. Data was elicited on a quarterly basis (four rounds in all) to capture seasonality, and the principal decision makers of the household – a male member and a female adult, were interviewed. Two measures of nutritional status, namely, Body Mass Index (BMI) (for adults) using anthropometric data and dietary diversity using the FAO prescribed dietary diversity score are analyzed in this paper to understand the effect of time use pattern on nutritional status. Time use patterns (using time diaries) are analyzed by gender with a 24-h recall methodology using a standard questionnaire which was adapted from the Women's Empowerment in Agriculture Index (WEAI, pilot 1) module. Labor use data from the input-output module of VDSA was analyzed to understand the time spent in agriculture by men and women, by operation. Data on access to toilets and drinking water was used in the analysis as these two variables influence nutritional outcomes. The cropping pattern analysis presented in this paper are only for the six villages of Telangana and Maharashtra. Box 1 presents a summary of the study locations and other details.

### 2.1 Conceptual and analytical framework

**Fig. 1:**
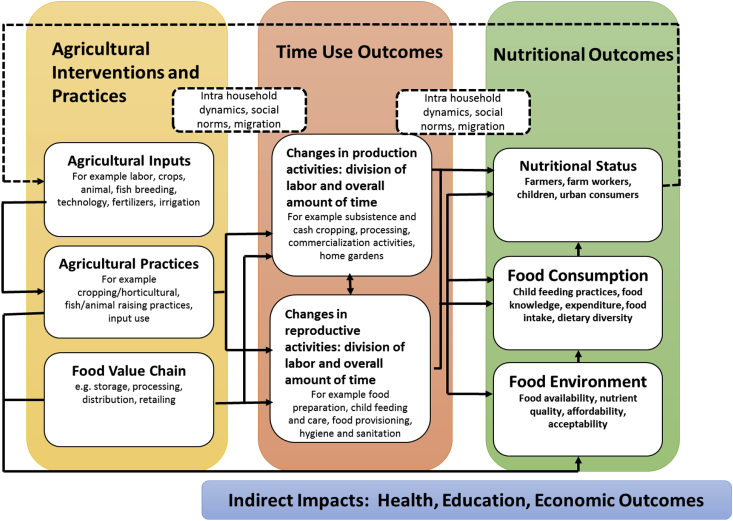
Conceptual framework linking agricultural practices, time use and nutrition outcomes.

*Source*: Fig. 2.1. in Johnston et al., (2015).

The theory of change set out by Johnston et al. (2015) has been used as the conceptual framework (Fig. 1). An examination and analysis of time use patterns of men, women and adolescent boys and girls in rural areas throws in-depth light on the inequalities at the household level, which could be social, cultural or economic and their impact on the different dimensions of well-being. Each pathway has the potential for varied or undesirable nutritional outcomes. It is reiterated that changes in time allocation and labor use are an output of varied agricultural practices and hence time use is an important variable in understanding nutritional outcomes. For example, a time use analysis might suggest that adding paid work outside the home on top of the existing ‘invisible’ workload inside the home would pose a significant burden on women, particularly poor women, in the vulnerable and marginalized regions of the SAT. Another example that explains the framework in the context of this paper is how changes in cropping pattern or agricultural practices have changed the gender division of labor and altered time demands on men and women and thereby their nutritional status. This calls for different sets of policies to address specific forms of burden management and time constraints shouldered by households, individual household members, or both, and interventions directed at women, especially from poorer and smaller households.

### 2.2 Analysis of the data (refer to Box 1 for variables)

A descriptive analysis of the data was complemented by a correlation scatter graph computed to understand the relationship between nutritional status, diet quality and sanitation and time use patterns across gender.^4^ The data was analyzed for statistical significance through a multivariate logistic regression to find the causal relation between individual nutritional status (with BMI as a proxy) and time use pattern along with other confounding/supporting covariates.

For the regression analysis, the variables were classified as follows.

• For a better analysis of the data, the activities from the 24-h time use recall were regrouped into composite activity groups upon referring to the literature on the energy costs of daily activities in rural India and based on the energy expenditure values (Kennedy, 2010 5; FAO, 2004). Based on Annex 5 of the FAO (2004) publication, all activities with a Physical Activity Rate (PAR) of less than 4.0 were considered as low energy consuming activities, and those with a PAR of 4.0 and above were considered as high energy consuming activities.
• BMI, the accepted measure of overall health in a person, was computed for in the respondents, whose anthropometric data was available. In this paper, the BMI (World Health Organization, 2015) of those in the 18–60 years age group was considered as per the accepted standard procedure.
• Dietary diversity scores which reflect diet quality were analyzed; the different food groups consumed were calculated, rather than the number of different foods consumed. The Individual Dietary Diversity Score (IDDS) (FAO, 2013) as a proxy measure of the nutritional quality of an individual's diet was modified by classifying the foods consumed into three groups, namely, calorie- and protein-rich foods; calorie-, protein- and micronutrient-rich foods and only calorie-rich foods.
• The other criteria used in the analysis was access to drinking water and sanitation, which was classified into four categories – access to both toilet and drinking water; no facility at home; access to only drinking water and access to only toilet. The composite activity groups and the BMI ranges used throughout the paper are classified in Box 2.
  Box 2 **Classification of activities as per energy consumption and BMI.**
  **Table:** 

Activities in the questionnaire	Activities in composite form	Activities as per energy consumption (Based on Annex 5 of FAO, 2004)
Pre-cooking work	Domestic work including food preparation	High energy consuming activities: Activities that have a Physical Activity Rate (PAR) of 4.0 and above
Cooking work		
Other domestic work		
Family care	Family care	
Farming livestock and other farm work	Farm, non-farm, livestock	
Non-farm work		
Personal care	Leisure (excluding sleeping)	Low energy consuming activities: Activities that have a PAR less than 4.0
Leisure and recreation		
Sleeping and resting	Sleeping and resting	
Other work	All others	
Travelling and commuting		
BMI ranges	Nutritional status	
<18.5	Underweight	
18.5 to 24.9	Normal	
25–29.9	Overweight	
>30	Obese
  Alt-text: Box 2

## 3 Results

### 3.1 Analyzing time use patterns from a diversity perspective: gender, social groups, age and activities

Time use is now increasingly used as a transformative indicator of gender equality. Time use analysis also reflects how gender roles attributed to women and men and girls and boys shape the division of labor within a household (Ferrant et al., 2014), a community and in agricultural activities/operations. Table 1a illustrates the time use by men and women in different daily activities in a typical day in the selected locations of the semi-arid tropics. The data is an average of four rounds collected during 2013–14. Compared to men, women spend more time on pre-cooking activities such as fetching firewood and drinking water, food preparation activities like cutting vegetables, cleaning grains, etc, cooking, other domestic chores and family care. Men spend more time on farming, non-farm and livestock activities as well as travelling, commuting and other miscellaneous activities. Women also participate in farm activities, as can be seen from the female/male ratio. The standard deviations (SD) are quite high in some cases and this is due to the wide range/scattered distribution by different members of the household with respect to time use. A plausible explanation for the high SD is explained in Table 2. A comparison of the total work hours (excluding recreation, leisure, sleeping and resting) across three developing countries – India, Bangladesh and Uganda is presented in Table 1b, leading to the conclusion that women in the Indian SAT spend an average of 177 and 105 min more compared to women from Bangladesh and Uganda respectively. Similar is the case with men, the difference is low compared to women. The harsh conditions of the SAT, the gender division of labor which is guided by social and cultural norms and prevailing agricultural practices/activities could be an explanation for these differences.

**Table 1a:** Gender-wise time use (in minutes) and magnitude of time use in different daily activities in the study villages.

Activities	Women		Men		Female/male ratio	
	Mean time in minutes (median value)	Standard Deviation (SD)	Mean time in minutes (median value)	Standard Deviation (SD)	Ratio	*t*-test
Precooking work	79 (65)	51	20 (4)	33	3.95	**
Cooking work	93 (94)	51	2 (0)	15	46.5	**
Other domestic work	62 (58)	36	3 (0)	14	20.67	**
Family care	44 (8)	77	6 (0)	23	7.33	**
Personal care	133 (131)	25	140 (139)	27	0.95	**
Farming and livestock	179 (124)	177	238 (182)	220	0.75	**
Non-farm work	82 (0)	131	179 (107)	199	0.46	**
Travelling and commuting	27 (15)	33	48 (38)	45	0.56	**
Recreation and leisure	174 (158)	127	232 (210)	146	0.75	**
Sleeping and resting	562 (548)	99	554 (548)	94	1.01	
Other work	6 (0)	43	18 (0)	70	0.33	**

**Table 1b:** Comparison of Total Work Hours^1^ across three countries (excluding recreation, leisure, sleeping and resting).

India's SAT	705	654	Based on Table 1a, analysis of the eight villages data is presented in this paper
Bangladesh	528	558	Source: Table 1a, Table 1b, Seymour et al., 2017.
Uganda	600	486	

1 Data generated using WEAI Module – pilot 1, 24 h primary activities only; ** 5% level of significance.

**Table 2:** Age group-wise time use (in minutes) in different daily activities in the study villages.

Activities	18–29 years	30–39 years	40–49 years	50 years and more	CV (%)
**Women**					
Pre-cooking work	91	73	79	72	11
Cooking work	90	107	99	67	19
Other domestic work	70	62	64	44	18
Family care	87	27	23	30	72
Personal care	137	132	131	132	2
Farming livestock and other farm work	87	241	183	199	37
Non-farm work	61	94	90	82	18
Travelling and commuting	21	32	28	28	16
Recreation and leisure	203	138	182	199	16
Sleeping and resting	575	535	561	588	4
Other work	20	1	1	1	162
**Men**					
Pre-cooking work	21	17	19	21	10
Cooking work	2	0.19	4	3	68
Other domestic work	2	0.11	3	4	74
Family care	7	6	2	10	53
Personal care	143	140	139	139	1
Farming livestock and other farm work	160	230	284	300	26
Non-farm work	199	209	168	103	28
Travelling and commuting	46	55	46	44	10
Recreation and leisure	264	231	216	236	9
Sleeping and resting	555	546	553	570	2
Other work	41	7	7	9	105

Source: Authors' own calculations are based on ICRISAT – Gender and Nutrition data (2013–14).

**Fig. 2:**
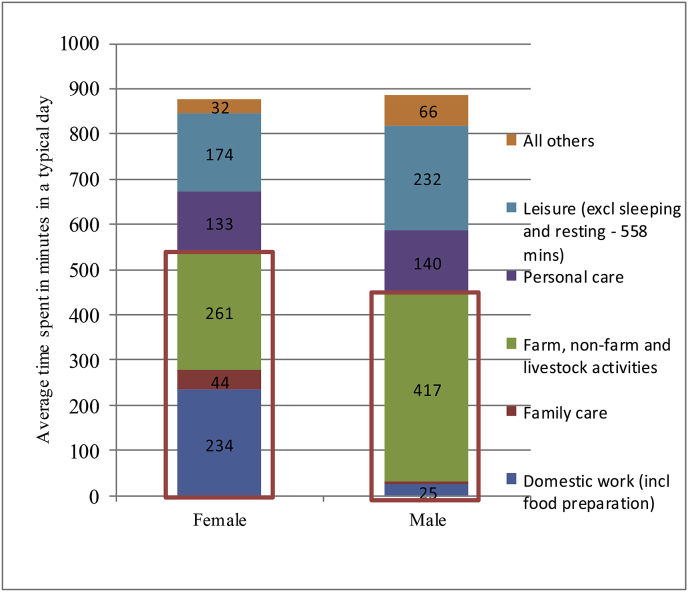
Gendered time use patterns in selected villages in rural SAT, 2013–14.

An examination of the overall time use patterns by gender using the classification of energy costs in activities reveal that compared to men, women spend a total of 539 min – about 2 h more than men – on activities requiring more energy (Fig. 2, see activities in red bracket). Similarly, they spent about 2 h less than men on leisure, personal care and other activities. In a typical day of 24 h, excluding sleeping and resting time, which constitutes a little more than 9 h for men and women, the latter reduced their leisure time by an hour and a half to accommodate the additional time demands on them for domestic, family care, farm and non-farm activities. In both Uganda and India, which have similar agro-ecologies, women's overall work burden is greater than that of men.

Younger women, especially those in the early years of child bearing (18–29 years), use more of their time on domestic, family care and personal care activities compared to women in the 40–49 and above 50 age groups, with a coefficient of variation as high as 72% among the different age groups (Table 2). Older women, ie, those 40 years and above, spend more of their time on economic activities including farm and non-farm work. Women in the 30–39 age group are the vulnerable group as they have to participate equally in the domestic sphere of the household as well as in the economic sphere. As a result, they compromise on family care activities (Table 2) which may lead to either improper care of the children and other members of the household or the redistribution of responsibilities within the family, like older siblings, especially girls, taking care of the younger siblings. On the other hand, irrespective of their age, men are engaged in economic activities (farm and non-farm) and participate less in the domestic sphere.

**Fig. 3:**
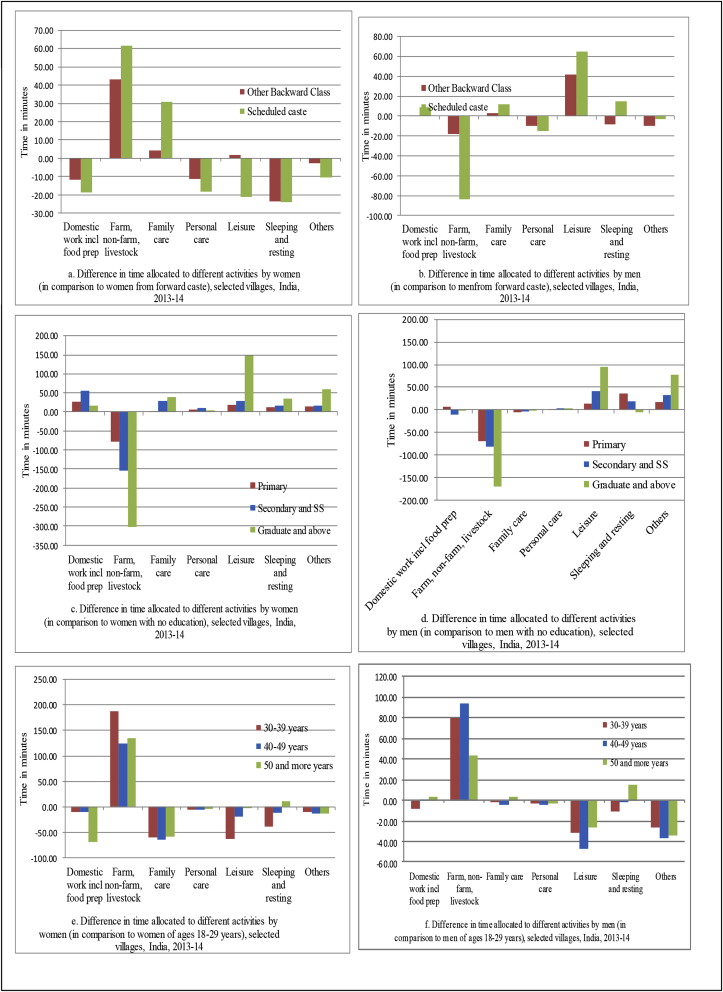
Effect of social group, education and age on time use, by gender, 2013–14.

Fig. 3 illustrates the effects of social group, education and age on time use by gender. Compared to women from Forward Castes (FC), women from Scheduled Castes (SC) and Backward Classes (BC) spent more time on farming, non-farm and livestock related activities. Women from the SC also spend more time on family care. They compensate for the additional time demands on these activities by cutting down their time on personal care, leisure, sleeping, resting and other activities (Fig. 3a). On the other hand, men from SC and BC spent less time on farming and related activities compared to men from the FC. One possible explanation for this is that more men from the FC are now involving themselves in farm supervision and working on their own farms because of the higher wage rates for men. Another explanation is that more men from the BC and SC are taking part in non-farm activities such as the Mahatma Gandhi National Rural Employment Guarantee Act (MGNREGA) which requires them to work for about 4 h a day and yet earn wages for the whole day Mishra (2014), Focus Group Discussions with the respondents). They spend less hours on farm/non-farm work and they have more time for leisure activities (Fig. 3b). As education level increases, both women and men spend less time on the farm and related activities and more time on domestic, leisure and other activities (Fig. 3c and d). Compared to men and women in the 18–29 age group, the analysis clearly indicates that both men and women in the 30–49 age group spend more time on farming, non-farm and livestock activities (Fig. 3e and f). Women cut down their time in domestic and care activities and leisure time to compensate for this workload. Younger men (18–29 age group) look for opportunities outside of farming and related activities. Young women, on the other hand, have more demands on their time with domestic, family care and other responsibilities at home as they are in the child bearing and rearing age group.

Similar results were documented by Connell (2013) for India, where for example, men devote 36 min to unpaid care responsibilities, out of which 36% goes into housework, with the remaining time spent on shopping, care of household members and travel related to household activities. Out of the 6 h women devote to unpaid care activities, the portion of time specifically spent on housework reaches 85%. Similarly, in a global context, compared to OECD countries, the gap between women and men in time spent on unpaid work in developing countries like India, Gautemala, Mexico and Mauritius ranges from around 3 h 30 min to 4 h 30 min (see Box 3).

Box 3 **Comparison of time spent on unpaid work in select developing and OECD countries.**

**Figure:**
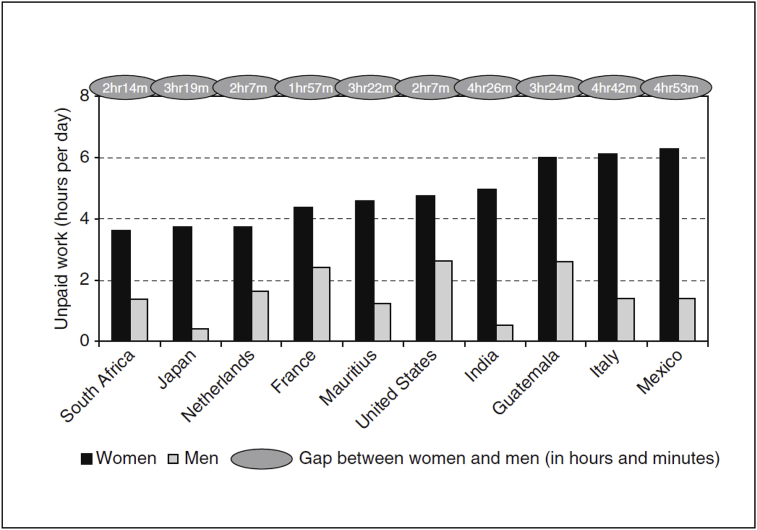

Alt-text: Box 3

*Source*: Antonopoulos, 2008, p. 9.

### 3.2 Gendered patterns of participation in agriculture: labor use

Data on labor participation also helps us understand and measure the amount of time spent by men and women on different tasks/activities/crops in agriculture. An analysis of the crop input and output module data from the VDSA villages between 1975 and 2014 reveals an interesting pattern. VDSA surveys collect time use data by agricultural operation, by gender over time. Confidence intervals and error margins were computed for the labor participation data which are presented in graphs 4 and 5. Interpretation of the results taking into account confidence bands and error margins reveals that the results are reliable and the pattern of sample means is an estimate of the corresponding pattern of population means in the study locations.

**Fig. 4:**
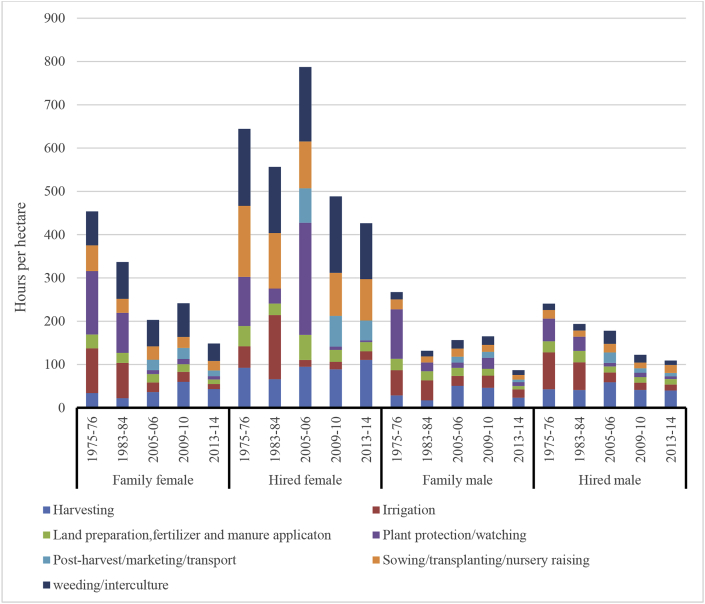
Labor use by operation and type of labor in select SAT villages, India.^6^.

An examination of the operation-wise participation of women and men in agriculture reveals that the involvement of women has increased over time not only in terms of the number of hours spent per hectare but also in the number of operations. In the early years (1975–76), women were involved mostly in sowing, weeding and harvesting; gradually over time, their role in land preparation, irrigation, plant protection and postharvest processing also became evident, so also an increase in their time burden (Fig. 4). Men, on the other hand, continue to perform the same operations over time and there is, in fact, a fluctuation in time use by men, which is declining. Even though the participation of women has increased in more activities, the overall labor burden of rural women lies in three major operations – sowing, weeding and related operations and harvesting while that of men is mostly in land preparation and related operations and irrigation. Men are also involved in harvesting nowadays because of the use of mechanization. Women spend around 75–94% of their time on sowing, weeding and harvesting while men spend 55–80% of their time on land preparation, irrigation and harvesting.

An examination of labor participation in agriculture – family versus paid/hired labor – presents an interesting picture. Fig. 4 shows an increase in time spent by female labor – both family female (FF) and hired female (HF) – from 1975 to 2014. However, the time by men – both family male (FM) and hired male (HM) – has been declining over the years, more so in the case of HM labor which has fallen drastically. Changes in cropping patterns in the study villages (e.g., from subsistence to commercial crops, intensification of agriculture, availability of irrigation, etc.) starting 2005 explain the increasing role of women in agriculture. The rising wages in agriculture (especially male wages) and the wage parity due to MGNREGA, has resulted in the deployment of family female labor in agriculture, especially during the peak season for sowing, weeding and harvesting of cotton. Women are spending on an average about 100–150 h per hectare on their own farms, which is a burden on their time, which use to be spent on domestic activities and family care. Their involvement in agricultural operations (against activities performed at home) also implies additional energy expenditure, and this has varying effects on their nutritional status.

Interpreting the above results taking into account the computed confidence bands and error margins confirms that women - both family and hired – are involved more in sowing, weeding and harvesting operations and the means presented in Fig. 4 fall within the confidence intervals. However, the error margins are varying over the years, by type of labor and agricultural operation. For example, in the case of harvesting operation by family female, the margin of error for the mean time spent was less than 5% during the years 1975–2005; while it was more than 5% during the years 2009 and 2013. A reverse trend in the error margin was observed for hired females for the same operation. For hired females, during the years 1975–2005, the error margin was high and it became smaller from 2009 onwards. This analysis thus shows that female time-use in agriculture varies by agrarian context, the crops cultivated as well as the extent of mechanization available and used, these are presented in Fig. 5a, Fig. 5b, Fig. 5c and discussed in detail in section 3.3. Overall, the operation wise analysis of time use in agriculture by labor type presents an understanding of the process of feminization of agriculture in the semi-arid tropics of India.

**Fig. 5a:**
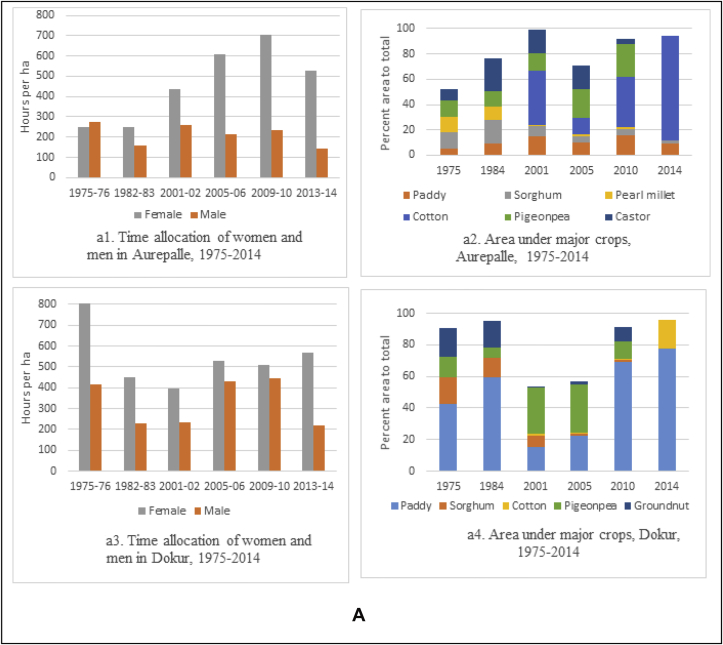
Gendered patterns of participation in agriculture, selected villages in Mahabubnagar district, 1975–2014. (refer footnote 6 for confidence bands).

**Fig. 5b:**
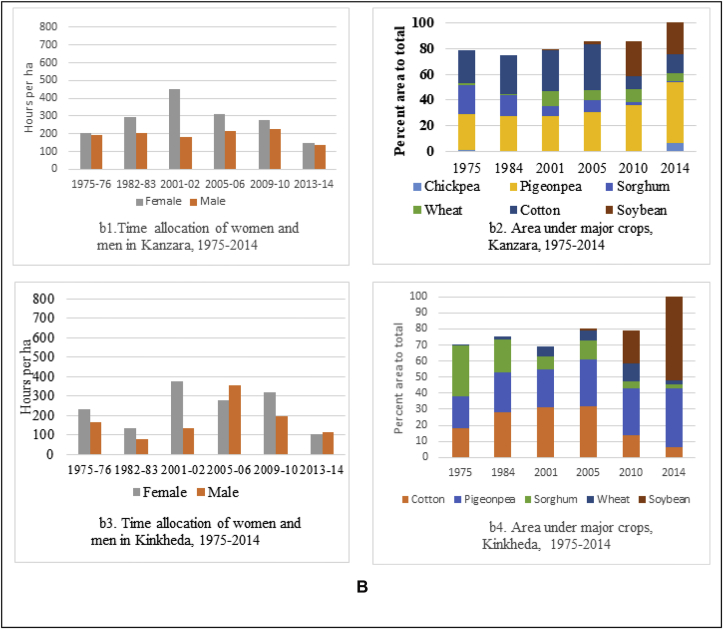
Gendered patterns of participation in agriculture, selected villages in Akola district, 1975–2014. (refer footnote 6 for confidence bands).

**Fig. 5c:**
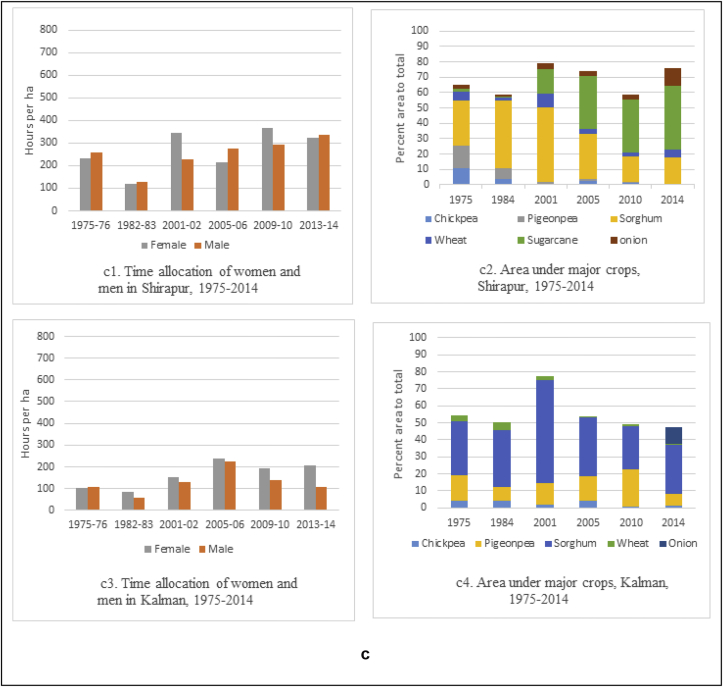
Gendered patterns of participation in agriculture, selected villages in Solapur district, 1975–2014. (refer footnote 6 for confidence bands).

### 3.3 Labor participation by cropping pattern

This paper analyzes cropping pattern and labor use data from 6 villages of Telangana and Maharashtra. Aurepalle and Dokur villages in drought-prone Telangana, where the pathway of development is through diversification of agriculture and diversification of income sources including migration, clearly shows that women are spending more time on agriculture as measured by the number of hours spent per hectare (Fig. 5a). Women's participation increased threefold from 258 h per hectare in 1975 to 840 h per hectare in 2010. This was due to changes in cropping pattern, from the dominating castor and sorghum in 1975 requiring less labor to the labor-intensive cotton starting the year 2010, requiring more female labor for harvesting/picking (Fig. 5a1 and 5a2). In Dokur, which once had a flourishing agriculture, experienced continuous dry spells and drought like conditions for a prolonged time (more than 8 years at a stretch). These conditions led to farmers shifting from paddy, the dominant crop in the 1970s and 80s, to castor during the dry spell years. The village also experienced migration by men and women to other towns and cities. The time allocation data and the cropping pattern data illustrate the declining participation of women in agriculture from 1975 to 2008/09. With good rains and a favorable environment post-2005, data shows that paddy cultivation has been increasing, leading to the increased role of men and women in agriculture (Fig. 5a3 and 5a4).

In Kanzara and Kinkeda villages in rainfall assured Akola, the pathway of development is through intensification of agriculture. Our analysis clearly showed that both men and women participated equally in agriculture as evidenced by their time allocation during 1975–1984/85 (Fig. 5b). During this period, diversification of agriculture was also observed (Fig. 5b1 and 5b2). Cotton, which has been grown here for centuries, is the dominant crop, and the area under it was increasing till up to 2003/04. During this period, the time use patterns of women showed an increased share in agricultural work. Starting 2007, soybean started gradually replacing cotton. The less labor-intensive soybean saw a drop in the time allocation of men and women (Fig. 5b1 and 5b2). Kanzara village uses more mechanization, which reflects more or less equal participation of men and women in agriculture. Similar trends were observed for Kinkheda as well (Fig. 5b3 and 5b4). In Kalman and Shirapur villages in the drought-prone region of Solapur with access to canal irrigation, almost equal participation of women and men in agriculture was observed (Fig. 5c). The cropping pattern in both these villages till 2001 was basically chickpea, pigeonpea and sorghum. With the availability of water for irrigation through the canal, the cropping pattern changed after 2001 to include more high value crops like sugarcane, vegetables and wheat in Shirapur (Fig. 5c1 and 5c2), while Kalman continued to grow the crops of the SAT and hence saw low participation of women and men in agriculture (Fig. 5c3 and 5c4). Kalman village has invested in the education of its children – both boys and girls; this saw a majority taking up teaching jobs in schools in nearby villages and towns.

To summarize, the sociological analysis from the long-term panel data from 1975 clearly points to evidence of a progressive increase in women's participation in agriculture in rural areas, although the extent varies across regions (Padmaja and Bantilan, 2013). The analysis reveals that in regions where agriculture is promising (eg, Kanzara) and favors sustained dependence on it, men and women jointly participate as they were doing since the early 70s. The role of women in agriculture increased in these cases but to a lesser extent. However, in regions (such as the Mahabubnagar villages) that have experienced shocks, women have a greater role in agriculture depending on the coping strategies the household adopts - changing cropping patterns and diversification; working as paid labor on others' farms and the migration of household's male members to towns, leaving the women to take care of the farms as well as participate in the care economy. This finding is echoed by Binswanger-Mkhize (2012) in his analysis of the structural transformation in India.

The analysis and the interpretations presented in sections 1, 2, 3 on the time use analysis based on a 24-h recall and participation in agriculture operations and activities add deeper insights into the heterogeneity in women's contribution in agriculture (SOFA team and Doss C, 2011). The sociological and descriptive analysis clearly bring out the fact that female time-use in agriculture varies by crop, production cycle, mechanization, wealth status and social group. The findings corroborate the earlier findings by different authors that sowing, weeding and harvesting are predominantly female activities and women contribute more than 75% of their time and labor for these activities. In total, at an aggregate level, the labour burden of rural women exceeds that of men, and includes a proportion of family labor who have other household responsibilities related to food preparation, participating in the care economy of the household and collecting fuel and water.

### 3.4 Time use, nutritional status and dietary diversity

Table 3 provides evidence to understand the effects of time demands on men and women in different activities on their nutritional status. First and foremost, it provides evidence of the magnitude of malnourishment by gender. Of the sample men, 62% have normal nutritional status and 38% are malnourished (of these, 23% are undernourished and 15% over nourished). Compared to this, only 54% of women are well nourished and 46% are malnourished. Focus group discussions with men and women in the study regions bought out opinions like - men who are underweight spend less time in high energy consuming activities – domestic work, farm, non-farm and livestock activities – and spend more time on leisure, sleeping and resting. The conversations also pointed that their poor nutritional status prevents them from indulging in more intensive activities and that they may be tiring easily. Hence they spend more time sleeping and resting compared to men who have normal BMIs. However, this has not been established statistically and hence it is stated as an opinion of the sample respondents.

**Table 3:** Time use, nutritional status and diversity in diets in the study villages, 2013–2014.

BMI category	Normal	Overweight	Underweight
**Men**			
Percentage of sample (N = 649)	62	15	23
**Time use (in minutes)**			
Domestic work including food preparation	24	29	22
Farm, non-farm, livestock	413	416	385
Family care	5	7	6
Personal care	140	140	139
Leisure (excluding sleeping)	239	235	241
Sleeping and resting	552	543	571*
All others	64	66	72
***Diversity in diets (percentage of individuals)***			
More calorie-rich foods	2	0	2
Calorie- and protein-rich foods	39	32	49
Calorie-, protein- and micronutrient-rich foods	60	68	49
**Women**			
Percentage of sample (N = 635)	54	21	25
**Time use (in minutes)**			
Domestic work including food preparation	234	227	232
Farm, non-farm, livestock	263	236*	251
Family care	44	43	46
Personal care	132	133	132
Leisure (excluding sleeping)	172	206*	171
Sleeping and resting	560	563	566
All others	32	29	39
***Diversity in diets (percentage of individuals)***			
More calorie-rich foods	2	2	2
Calorie- and protein-rich foods	39	41	42
Calorie-, protein- and micronutrient-rich foods	59	58	56

Note: * indicates the deviation of average time use for different activities of normal BMI group individuals compared to either overweight group individuals or underweight group individuals were significantly different from zero at 5% level of significance.

The picture for women is different. The analysis reveals that in spite of being underweight and having a BMI of less than 18.5, women are more involved in high energy consuming activities compared to over nourished women. Moreover, they spend less time on leisure and personal care and this further affects their nutritional status. Third, on diversity of diets or diet quality, the findings suggest that there is no difference in the diet quality of men who are normal or malnourished. In the case of women, it is surprising to note that women who are undernourished have the most diversity in diets compared to normal or overweight women, which is a matter for further study.

### 3.5 Linking time use, nutrition and dietary diversity: A statistical analysis

In understanding the link between time use and nutrition outcomes, two types of analyses have been attempted in this paper. First, a scatter plot was plotted to understand this link. A multivariate regression analysis was also done to identify the variables that are significant in this link. Nutrition is a complex issue and does not depend only on diet quality and diversity. Utilization of the nutrients by the body is also important; so are access to and use of safe drinking water and sanitation facilities (toilets in this case). These two variables are included in the analysis.

#### 3.5.1 Correlation scatterplot

**Fig. 6:**
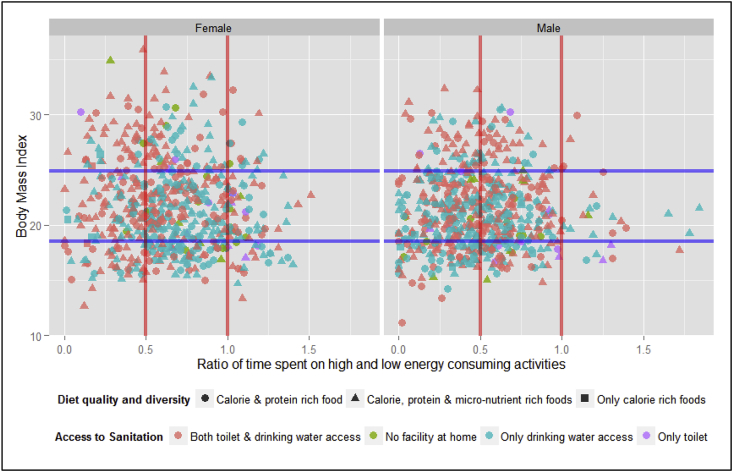
Correlation between time use and nutrition using a scatter plot, study villages, 2013–14.

The scatter plot showing the relationship between time use represented as a ratio of time spent on high energy consuming activities and low energy consuming activities and BMI with sanitation access and diet quality as additional variables reveals that both undernutrition and over nutrition are more prevalent in females than in males (Fig. 6). Women tended to be undernourished when they were spending more time in activities that required high energy. When the sanitation and diet quality variables were overlaid on this analysis, it can be seen that individuals – both men and women – are malnourished in spite of having access to both drinking water and toilets and having a diverse diet with foods rich in calories, proteins and micronutrients (the orange triangles in the plot). This analysis confirms that mere access to safe drinking water and toilets is not a sufficient condition to improve nutrient utilization by the body. Good hygiene and better sanitary practices have to be adopted by the rural people. A limitation of this analysis is that dietary diversity scores inform only about the diet quality; the quantity consumed is not understood from this indicator. From the scatter plot, it can be inferred that the triple burden of malnutrition is seen emerging in rural India as well. While the quality of diets has improved, access to sanitation facilities has improved and women (compared to men) are spending more time in activities requiring high energy, there is an observed increased overnutrition in women. This could be due to the consumption of insufficient quantities (as per the recommended dietary allowances) of foods rich in micronutrients (leading to anemia, calcium deficiency) or increase in consumption of foods rich in calories and proteins (leading to overweight and increase in lifestyle disorders like cirrhosis, type 2 diabetes, etc.). Both overnutrition and undernutrition conditions could be due to improper utilization of the nutrients by the body because of enteric organisms and infections (non-use of toilet and improper storage and handling of drinking water).

#### 3.5.2 Multivariate regression analysis

To establish and understand the relationship between individuals’ time use in different activities and other covariates and influence on nutrition outcomes, a multivariate logistic regression analysis was performed (Table 4). The analysis is presented in comparison with individuals with good nutritional status (normal BMI). It can be inferred that there is a significant relationship between time spent on high energy consuming activities and underweight but an inverse relationship with overweight which is highly significant. Overweight individuals spend considerably less time on activities requiring higher energy consumption. When the time use variable is analyzed together with an interaction variable (age), the relationship is highly significant for both underweight and overweight categories of individuals. Women tended to be more overweight than men even though they were spending more time in energy consuming activities than men. This is because of the changes in their diet composition. The diets are primarily rich in carbohydrates; sorghum which was the dominant cereal food is replaced by rice. Rice is easy to cook, available at a subsidized price through the Public Distribution System and is consumed in larger quantities to reach a state of satiety compared to sorghum. Thus. instead of losing weight, women are gaining weight.

**Table 4:** The relationship between individuals’ time use in different activities with other covariates and individual nutritional status: A logistic regression analysis.

Variables	Underweight Vs Normal BMI	Overweight Vs Normal BMI
	Coefficient	Coefficient
Constant	0.2266	−0.8671
Time spent on high energy consuming activities (24 h' time frame)	0.0715*	−0.1469***
Interaction variable (Time use in high energy consuming activities * Age)	−0.0027***	0.0034***
Gender dummy (Male = 1, Female = 0)	−0.2087	−0.5301***
Education level (Illiterate = 1, Primary = 2, Secondary & Senior secondary = 3 & Graduate and above = 4)	−0.0915	0.1436
Diet quality and diversity (Only calorie-rich foods = 1, calorie- and protein-rich foods = 2 and calorie-, protein- and micronutrient-rich foods = 3)	−0.1731	−0.0585
Access to sanitation (No facility at home = 1, only toilet = 2, only drinking water access = 3 and both toilet & drinking water access = 4)	−0.1734	0.1709
Log per capita income	−0.0501	0.033
Food and non-food expenditure ratio	0.079	−0.1435
Caste dummy OBC (Forward caste is the reference)	0.4431***	−0.4481***
Caste dummy SC (Forward caste is the reference)	0.4508***	−0.4064
Region dummy Maharashtra (Andhra Pradesh region is the reference region)	0.6758***	−0.7561***
Region dummy Telangana (Andhra Pradesh region is the reference region)	0.4564***	−0.8746***
		
LR chi square	36.61	53.36
Prob > chi2	0.00	0.00
Pseudo R2	0.03	0.05
Log likelihood	−576.61	−484.16
Number of observations	992	929

Note: ***p < 0.01, **p < 0.05, *p < 0.1.

Education is important in enhancing nutritional status but it did not show up as a significant variable. Caste and region were highly significant variables in influencing nutritional outcomes as dietary habits and patterns are strongly influenced by social and cultural norms which vary by social groups and region.

### 3.6 Robustness test of regression model

To estimate the efficiency of the model and accuracy test, Hosmer-Lemeshow test was performed (Lemeshow and Hosmer, 1982; Hosmer et al., 2013). The test statistics - p-values for model-1 (Underweight Vs Normal BMI) and model-2 (Overweight Vs Normal BMI) were 0.532 and 0.602 respectively, indicating that both the models have a reasonable good fit and efficient to predict the model appropriately.

## 4 Discussions

The descriptive analysis and the rich narrative bring out several findings related to women's work and their overall time use, including care giving activities. Women are equally engaged in productive work, reproductive and domestic work. The reproductive activities which are in the form of unpaid labor in their own homes tend to not be accounted for, even by women themselves. Family and personal care are taken together as time spent only on child care is difficult to account for by the women themselves. Women are engaged in the care of not just the children but also other members of the family, like the elderly and the sick. Also, sometimes there are multiple child care providers and women also multi-task child care with their other activities, a finding which is very similar to Jain and Zeller (2015) in Bangladesh. These micro-level insights indicate that women do not reduce their time in cooking and household chores even though there is high demand on their time due to agricultural intensity. Instead, they cut down time on activities which can be substituted by other members of the household and family.

The findings are echoed by Hirway (2009) illustrating that poor women seem to be spending relatively more time in the management of households doing cooking, cleaning, washing and taking care of household members, especially children, the sick and elderly. The time spent thus is significant in both developed and developing countries. Hirway and Jose (2011) further elucidate that compared to developed countries, much more time is likely to be spent on these activities by the poor in developing countries. This could be due to inadequate public infrastructure/public provisioning of the basic needs in developing countries forcing the cash-poor households to access these facilities through free collection or within the home and not buy in the market; or the technology used by the poor is likely to be of a lower standard, demanding more time and more efforts.

Social groups and income influence the way women spend their time, including leisure time. Time spent by women outside the home either for paid work or unpaid work does not affect their food consumption or that of their children. In some ways it improves the diversity in diets as they become more aware of their eating patterns and habits. Insights from the field do not support the hypothesis put forward by literature, that child care practices and/or dietary quality are constrained by the time women spend on preparing food or their overall work outside the home.

The descriptive narratives from the data on time spent in agriculture and related activities, informs us that compared to men, or over the years, women spend almost double the number of hours per hectare on agriculture activities either on their own farms or as paid labor. Sowing, weeding, harvesting continue to be the dominant activities for which women spend about three-fourths of their time in agriculture. Further inferences are that in the event of rising wages in the rural areas, women (especially family female) substitute for male labor and perform activities performed by men. The time-use analysis and the changing cropping patterns in the rural areas of the SAT also indicate that activities performed by men are mechanized, freeing them from agriculture into off farm or non-farm activities, including migration to towns and cities. The activities that women perform dominantly on the crops that continue to be grown in these areas are not always mechanized, women participate more in agriculture due to migration of the economically active men and hence there is seen greater participation of women in agriculture in these regions. The narratives provide the evidence of changes in time use by gender, over time and how this is associated with changes in cropping patterns. Time use data clubbed with the analysis of the anthropometric data and the dietary diversity data provides the association with the changes in adult BMI over the years to time use and participation in agriculture.

The rich narratives presented in the paper point to the evidence that India is facing the triple burden of malnutrition. This is because of the continued dependence on starchy cereals and staples to meet energy, protein and micronutrient needs. Diets have to become more diverse not just in terms of quality, decreasing the consumption of carbohydrate-rich foods while increasing the consumption of protein- and micronutrient-rich foods. These include high value commodities like green leafy vegetables, fruits, other vegetables, milk, meat and other livestock products. The health sector also needs to recognize this fact and respond to the problems associated with both undernutrition and overweight.

Good sanitation is important for the absorption of nutrients by the body. Open defecation common in rural India and unhealthy sanitation practices can lead to undernutrition. Enteric infections have a clear effect on weight loss, loss of key micronutrients and the non-absorption of key nutrients into the blood resulting in underweight and other associated symptoms and effects. Enteric infections can also lead to chronic inflammatory responses, inhibited protein synthesis and malabsorption of nutrients, among others, that are all associated with obesity. There should be a strengthened focus on diet quality and safe hygienic practices in relation to malnutrition.

A behavior change campaign and education are key to creating awareness about healthy eating and good sanitation practices. Government programs and policies should target the provision of nutri-rich cereals through the Public Distribution System and also provide incentives to grow crops such as sorghum, millets and legumes. Empowering women with more information and control over household income will enable them to make decisions towards efficient time use, food consumption, sanitation and healthy practices.

## 5 Conclusions

The sociological analysis clearly points out that men and women have different time use patterns and burdens. Time matters, especially for women who are involved in agriculture and also have to care about the household economy. These include poor women, women in the child bearing age and the vulnerable. The paper concludes that time commitments – either increased or reduced – have a complex impact on the nutritional outcomes of women and children. The rich narratives point towards this evidence. The varied outcomes necessitate different sets of policies and innovative approaches to leverage agriculture for nutrition from a gender-responsive perspective. While acknowledging that agriculture is important for women, there is a need to strengthen theirs access to the resources needed for productive agriculture, and reduce the time and energy burdens of household work including food processing and preparation (Doss et al., 2018). To this effect, improved methods to measure empowerment are needed so that linkages between increasing resources controlled by women and nutrition as well as linkages between women's empowerment and nutrition are established empirically.

## Footnotes

1 Earlier known as the ICRISAT Village-level Studies (VLS) running from 1975; http://vdsa.icrisat.ac.in/for more details; funded by the Bill & Melinda Gates Foundation for the period 2009–2014.

2 Funding support from the CGIAR Research Program on Policies, Institutions and Markets (CRP–PIM) with a focus on nutrition and women's empowerment.

3 The data collection tools and instruments are available on http://vdsa.icrisat.ac.in/.

4 The analyses in the tables and figures (except Fig. 1) are the author's own calculations based on ICRISAT - gender and nutrition data (2013–14) and VDSA data (1975–2014).

## Footnotes

5 The classification was done based on notes prepared by Gina Kennedy on measuring energy expenditure and physical activity; see http://www.a4nh.cgiar.org/files/2015/01/Energy-Expenditure-and-Physical-Activity-Reference-Notes1.pdf.

6 **Table:**

Note for Fig. 4, Fig. 5a, Fig. 5b, Fig. 5c: The confidence bands and error margins were computed by the authors at 95% level of confidence for each operation and labor type.								
Type of labor →	Family female		Family male		Hired female		Hired male	
Operation ↓/Confidence band →	Lower	Upper	Lower	Upper	Lower	Upper	Lower	Upper
Harvesting	34.72	51.18	28.98	38.55	88.58	121.74	42.24	63.17
Irrigation	20.44	80.91	40.63	55.90	6.06	136.85	25.64	61.57
Land preparation, fertilizer and manure applicaton	17.85	25.73	16.17	20.23	26.68	43.33	17.67	24.34
Plant protection/watching	27.35	85.97	22.60	49.45	18.13	181.05	10.82	37.64
Post-harvest/marketing/transport	17.64	24.34	11.40	15.32	46.22	77.22	12.94	19.15
Sowing/transplanting/nursery raising	26.54	44.53	13.48	18.15	101.15	145.99	14.95	20.37
weeding/interculture	55.34	75.85	17.87	26.10	139.85	179.71	15.26	29.56
